# A time series study on the effects of heat on mortality and evaluation of heterogeneity into European and Eastern-Southern Mediterranean cities: results of EU CIRCE project

**DOI:** 10.1186/1476-069X-12-55

**Published:** 2013-07-03

**Authors:** Michela Leone, Daniela D’Ippoliti, Manuela De Sario, Antonis Analitis, Bettina Menne, Klea Katsouyanni, Francesca K de’ Donato, Xavier Basagana, Afif Ben Salah, Elsa Casimiro, Zeynep Dörtbudak, Carmen Iñiguez, Chava Peretz, Tanja Wolf, Paola Michelozzi

**Affiliations:** 1 Department of Epidemiology, Lazio Regional Health Service, Via di Santa Costanza 53 00198, Rome, Italy; 2 Department of Hygiene and Epidemiology, Medical School, University of Athens, Athens, Greece; 3 WHO Regional Office for Europe, Climate change, sustainable development program, Bonn, Germany; 4 Spanish Consortium for Research on Epidemiology and Public Health (CIBERESP), Madrid, Spain; 5 Centre for Research in Environmental Epidemiology (CREAL), Barcelona, Spain; 6 IMIM (Hospital del Mar Research Institute), Barcelona, Spain; 7 Laboratoire d’Épidémiologie Médicale, Institut Pasteur de Tunis, Tunis, Tunisia; 8 Faculdade de Ciências da Universidade de Lisboa, Lisbon, Portugal; 9 Koç University, School of Health Sciences, Istanbul, Turkey; 10 Center for Public Health Research (CSISP), Valencia, Spain; 11 University of Valencia, Valencia, Spain; 12 Sackler School of Environmental research, Tel-Aviv University, Tel-Aviv, Israel

**Keywords:** Hot temperature, Mortality, Mediterranean region, Heterogeneity, Age groups

## Abstract

**Background:**

The Mediterranean region is particularly vulnerable to the effect of summer temperature.

Within the CIRCE project this time-series study aims to quantify for the first time the effect of summer temperature in Eastern-Southern Mediterranean cities and compared it with European cities around the Mediterranean basin, evaluating city characteristics that explain between-city heterogeneity.

**Methods:**

The city-specific effect of maximum apparent temperature (Tappmax) was assessed by Generalized Estimation Equations, assuming a linear threshold model. Then, city-specific estimates were included in a random effect meta-regression analysis to investigate the effect modification by several city characteristics.

**Results:**

Heterogeneity in the temperature-mortality relationship was observed among cities. Thresholds recorded higher values in the warmest cities of Tunis (35.5°C) and Tel-Aviv (32.8°C) while the effect of Tappmax above threshold was greater in the European cities. In Eastern-Southern Mediterranean cities a higher effect was observed among younger age groups (0–14 in Tunis and 15–64 in Tel-Aviv and Istanbul) in contrast with the European cities where the elderly population was more vulnerable. Climate conditions explained most of the observed heterogeneity and among socio-demographic and economic characteristics only health expenditure and unemployment rate were identified as effect modifiers.

**Conclusions:**

The high vulnerability observed in the young populations in Eastern-Southern Mediterranean cities represent a major public health problem. Considering the large political and economic changes occurring in this region as well future temperature increase due to climate change, it is important to strengthen research and public health efforts in these Mediterranean countries.

## Background

The Mediterranean basin is one of the areas of the world most influenced by current and future climate change, that is already affecting mortality and morbidity related to temperature increase during warm season [1,2]. Health effect estimates of high temperatures are available only for the European countries surrounding the Mediterranean region [3-5]. Evidence from these multicenter studies suggests a variability in the temperature threshold levels above which summer temperature has an impact on mortality and in the slope of the curve above this value. Local climatic conditions are an important determinant of such heterogeneity; higher thresholds and higher effects were generally found in populations that are more often exposed to extreme summer temperatures [3,5].

On the contrary no specific evaluation has been carried out in non-European Mediterranean areas, including North Africa and Middle East countries, usually exposed to more extreme temperatures during summer with respect to the other Mediterranean countries. In fact, summer conditions may vary between different regions of the Mediterranean basin, with prolonged very hot and dry summers along the north African coast and middle eastern cities, and slightly milder and shorter summers in southern Europe and some areas of Turkey.

Socio-demographic characteristics of Southern Mediterranean countries are largely different from European countries with a greater proportion of young population, higher infant mortality rates, and high illiteracy rates in the working age populations, especially among women due to cultural and religious restrictions [6]. Moreover, population in these countries is rapidly growing, but this is not paralleled by the economic growth that is hampered by their totalitarian regimes [6]. Consequently per-capita GPD indicator in these countries is well below the European ones with therefore limited resources in the health care system that result a low adaptive capacity to current climate conditions and climate change. In contrast in European countries a great amount of economic resources is devoted every summer to put into effort heat prevention plans [7,8].

Considering the large differences in terms of climate, socioeconomic and demographic characteristics between countries surrounding the Mediterranean basin, it could be expected that these characteristics may modify the effect of summer temperature leading to a large heterogeneity in the health impact across populations. However, the contribution of demographic and socioeconomic characteristics has not evaluated yet in the Mediterranean region. The only available evidence comes from US studies reporting population density [9,10], race [9-11], unemployment [10], and air conditioning prevalence [9,10,12], as modifiers of the relationship between temperature and mortality during summer. Regards other socioeconomic indicators such as education level and income, results are scarce and more controversial [10-12], despite these indicators at both individual and community level are important determinants of a populations’ adaptive capacity especially in low and middle income countries [13].

This study evaluated the effect of summer temperature in the Mediterranean region including for the first time Eastern-Southern Mediterranean cities, from North Africa and the Middle East. The study hypothesis is that summer temperature has an effect on mortality in these cities and that effects may be different from those observed in European cities around the Mediterranean. Since the European and Eastern-Southern Mediterranean countries are characterized by different climate conditions, demographic and socioeconomic structure and health services organization, we hypothesize that these city characteristics may have a role as determinants of between-city heterogeneity. The study is part of the CIRCE project, an EU funded project carrying out an assessment of the climate change impacts in the Mediterranean and was carried out under Research Line 9 “Human Health”.

## Methods

### Study design and population

A time-series approach was followed to estimate the effect of summer temperature on mortality in 10 cities around the Mediterranean: Athens, Barcelona, Bari, Istanbul, Lisbon, Palermo, Rome, Tel-Aviv, Tunis and Valencia and to evaluate the between-city heterogeneity. Although located outside the Mediterranean basin, Lisbon was included as it lies within the Mediterranean climatic zone. The study population includes total population resident in the study cities, for an amount of about 20 million people. Time series data included years from 1991 to 2007 with at least three consecutive years for each city. The study period was restricted to the summer season (April-September) being the focus of the analysis on the effect of summer temperature.

### Mortality data

For each city, daily mortality data were obtained from local mortality registries. Daily deaths among resident population for all natural causes (ICD-9: 1–799) were considered for the following age groups: 0–14, 15–64, 65–74, 75+ years, and all ages combined. Istanbul and Tel-Aviv data were only for an old period and even the age groups 0–14 years and 75+ years were not available in Istanbul.

### Exposure variable

For each city, meteorological data collected every 6-hours, were obtained from airport weather stations located closest to the city. As exposure variable maximum apparent temperature (Tappmax) was chosen *a priori* to better measure the physiological discomfort due to the combined effect of temperature and humidity than temperature alone. Apparent temperature was calculated according to the formula proposed by Kalkstein [14], and adapted by O’Neill [11]:

$$\mathrm{AT}=-2.653+0.994\times \mathrm{temp}+0.0153\times {\left(\mathrm{dew}\right)}^{2}$$

For Istanbul, due to the lack of data, the mean apparent temperature was calculated. A delayed effect of Tappmax up to 3 days (Tappmax averaged for day 0 and previous 3 days) was considered for all cities, based on an exploratory analysis throughout Distributed Lag Models (data available on request).

### City characteristics

Several characteristics at city level were considered as potential factors explaining the geographical heterogeneity in the effect of summer temperature among cities. Socio-demographical, health care and geographical indicators were retrieved from: the World Bank [15], Organization for Economic Cooperation and Development [16], WHO [17-19], and National Institute of Statistics websites (Additional file 1: Tables S1-S2). The city characteristics considered in the study include determinants of population susceptibility (i.e. percentage of elderly population, percentage of unemployed, life expectancy at birth, infant mortality rate and population size) and resources for adaptation (GDP per capita, health expenditure and hospital beds density). Variables describing local climatic conditions during the summer were also considered (Mean, maximum, minimum and standard deviation of temperature and of Tappmax, mean and standard deviation of relative humidity, Latitude and Longitude).

### Statistical analysis

The analysis was developed in two steps, first a common model was applied to each city to obtain city-specific estimates, then results were combined in a random effect meta-analysis to explore the role of potential effect modifiers of the Tappmax-mortality relationship.

### City-specific model

The city-specific association between Tappmax at lag 0–3 and mortality was assessed by Poisson Generalized Estimation Equations (GEEs), assuming a linear threshold model. The effect of summer temperature was modeled as log-linear increase in risk above the threshold. The city-specific effect estimates of relative risks of dying for 1°C increase of Tappmax (lag 0–3) above the threshold were expressed as percent changes. Moreover, percent deaths attributable to temperatures above Tappmax threshold were calculated from relative risks [20]. The analyses were repeated separately for each age groups.

#### Model specification

The modeling choice was driven by two main considerations: the first regards the shape of the temperature-mortality relationship and the second deals with the choice of a model able to take into account the autocorrelation structure within data. As for the first aspect, a linear threshold model was assumed to be a good approximation of a more complex non-linear model [3,12,21,22]. The strength of the linear threshold model assumption was checked in a flexible way using Poisson Generalized Additive Models (GAMs) [23]. GAMs was performed using a cubic regression spline for the exposure with one knot every 8°C [3]. The visual inspection of city-specific Tappmax-mortality relationships justified the choice to model the non-linearity of curves by introducing two linear terms below and above the threshold. Threshold was interpreted as Tappmax value above which mortality begins to increase and were estimated following the segmented linear regression for unknown break points proposed by Muggeo [24]. Where multiple thresholds may be identified by the Muggeo algorithm, the threshold corresponding to the highest Tappmax value was selected. The segmented regression models for specific age groups (0–14, 15–64, 65–74, 75+) were unstable due to limited mortality counts, hence the break points were estimated only for the all ages population.

A sensitivity analysis was run with different starting points to test the robustness of thresholds identification by Muggeo algorithm.

The second aspect driving the model choice is linked to the structure of the data where the observations are restricted only to the summer months allowing to take into account the serial correlation within each summer period and assuming independence between summers.

Specifically a first order autoregressive structure for the correlation within each summer was defined a priori based on the previous studies that involved most of the cities included here [3,4]. Furthermore in a sensitivity analysis the best working correlation structure was checked following the quasi-likelihood under independence model criterion (QIC) [25].

Model based variance estimator for the standard errors was used as recommended in the presence of few large clusters [26].

#### Confounders

The following variables were included as potential confounders: barometric pressure, wind speed, 24-hour mean NO_2_, holidays, day of the week and calendar month and time to control for seasonality and long time trend respectively. Barometric pressure and wind speed were included as linear terms according to previous studies [3,4]. In a sensitivity analysis, barometric pressure was also considered as a smooth term. For this variable the same lag as Tappmax was considered. According to previous studies, NO_2_ was considered as indicator of traffic-related air pollution that is the dominant source of air pollution in urban areas. NO_2_ data were not available for Istanbul and Bari. Other pollutants were not investigated as potential confounders of the Tappmax-mortality association (i.e. ozone, PM10) because of the unavailability of data.

Holiday, day of week and calendar month were introduced as indicator variables. Time was included as linear and quadratic term. A sensitivity analysis on alternative modeling choices for controlling seasonal and long time trends was carried out.

### Meta-regression analysis

City-specific estimates were included in a random-effects meta-analysis to assess between-city heterogeneity using the maximum likelihood method in the *metareg* procedure in Stata [27]. Then socio-demographical, geographical and health care indicators at city level were considered as potential effect modifiers explaining heterogeneity. After having run the model without covariates, each city characteristic was introduced in the model singularly to test its role as effect modifier in terms of explained between-city variance in agreement with other studies [9,13]. The city characteristics which explained more than 10% of between-city variance and with statistical significance at 80% level (p-value<0.2) of meta-regression coefficients were considered as effect modifiers.

Results were shown as predicted percent change in mortality at the 25^th^ percentile and 75^th^ percentile values of the effect modifier distribution [9]. Meta-regression analysis was carried out for all ages.

To evaluate the robustness of meta-regression results and therefore possible bias in the effect estimates with respect to the choice of temperature threshold values, a sensitivity analysis was performed using city-specific estimates calculated on a more extreme temperature threshold (95^th^ percentile).

## Results

Table 1 describes demographic, socioeconomic and health care characteristics of the study cities.

**Table 1:** Demographic, socioeconomic and health care characteristics of study cities

**City**^**a**^	**Demographic indicators**						**Socioeconomic and health care indicators**^**c**^			
	**Total population**^**b**^	**Ages 0-14 (% of total)**^**b**^	**Ages 15-64 (% of total)**^**b**^	**Ages 65 + (% of total)**^**b**^	**Infant mortality rates (per 1000 live births)**^**c**^	**Life expectancy at birth (years)**^**c**^	**Hospital beds density x 1000 inhabitants**	**GDP per capita (US$)**	**Health expenditure (% of GDP)**	**Unemployment rate (% of total labor force)**
Rome	2,546,804	12.8	68.1	19.1	4.6	80.1	4.6	19,722	8.2	9.6
Barcelona	1,505,325	11.5	66.5	22.0	4.1	79.7	3.6	14,952	7.2	10.5
Bari	316,532	14.1	68.7	17.2	4.6	80.1	4.6	19,722	8.2	9.6
Istanbul	8,803,468	25.8	69.4	4.8	27.8	71.5	2.6	3,037	5.2	8.4
Valencia	738,441	12.8	69.7	17.5	4.1	81.2	3.6	14,952	7.2	10.5
Lisbon	564,657	11.6	64.8	23.6	5.0	79.7	3.9	11,691	8.8	4.0
Palermo	686,722	17.5	67.8	14.7	4.6	80.1	4.6	19,722	8.2	9.6
Athens	3,894,573	14.3	70.9	14.8	5.1	78.5	4.8	11,858	8.8	10.2
Tunis	983,861	21.3	71.9	6.8	21.0	72.8	1.7	2,281	5.8	15.1
Tel-Aviv	348,245	17.9	63.7	18.3	5.1	79.5	4.2	19,093	7.8	9.3

^a^ Cities are ordered by latitude.

^b^ City level. Source: National Institute of Statistics.

^c^ National level. Source: OECD Statistics extract. WHO, World Health Statistics. World Bank, World Development Indicators.

Cities differ greatly in population size, from around 9 million inhabitants in Istanbul to around 300 thousand inhabitants in Bari and Tel-Aviv. Population age structure shows a different pattern among European, North African and Middle Eastern cities, with a lower percentage of people aged over 65 in Tunis and Istanbul (below 10%). Turkey and Tunisia have a higher infant mortality rate (greater than 20/1000 live births) and a lower life-expectancy at birth (about 70 years) with respect to the other Nations. These countries also have the lowest hospital bed density (<3/1000 residents). The national GDP per capita divides the countries into three groups: Italy Israel and Spain with GDP around 15,000-20,000 US$, Portugal and Greece with GDP around 10,000 US$ and Turkey and Tunisia with GDP around 2,000-3,000 US$. Regarding health expenditure, a similar pattern to GDP can be observed, with Turkey and Tunisia having only 5% of GDP devoted to health care. A different pattern was observed for unemployment indicator, where Tunisia and Spain having the higher rates.

Concerning exposure, Tappmax showed great variability among cities, with lowest mean values observed in Barcelona and Lisbon, and the highest values registered in Tel-Aviv, Valencia, Tunis and Athens (Additional file 1: Table S3). Similar patterns were observed in terms of mean, maximum and minimum air temperature; North-African and Middle East cities recorded the highest value. There was large heterogeneity among cities in terms of relative humidity with Istanbul recording the highest value (75.2%). Also a great heterogeneity was observed in terms of Tappmax thresholds; the highest value are reported in Tunis (35.5°C), Tel-Aviv (32.8°C) and Valencia (32.0°C).

**Figure 1:**
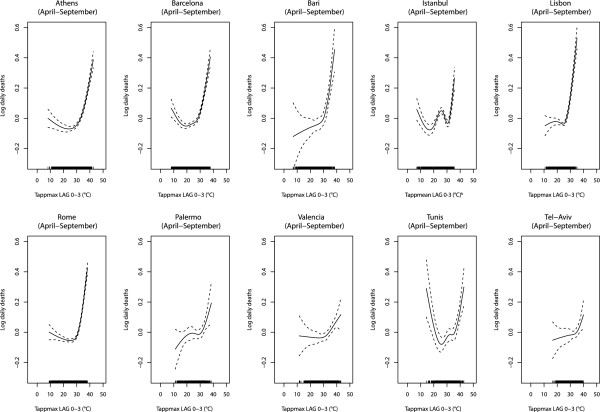
**Tappmax-mortality relationship**^**a **^**for all causes and all ages during summer season (April-September), **^**a **^**GAMs adjusted for potential confounders: barometric pressure, wind speed, 24-hour mean NO**_**2**_**, holidays, day of the week, calendar month and long time trend, **^**b **^**Tappmean: mean apparent temperature.**

Figure 1 shows city-specific exposure-response curves of the relationship between Tappmax and daily mortality for the all ages population. A clear J-shaped relationship can be observed in Athens, Barcelona, Bari, Rome, Palermo and Lisbon while in Tunis and Istanbul the curves assumed a more U-shaped form. In Tel-Aviv and Valencia the relationship seems less steep respect to the other cities. From a visual inspection, the temperature value above which mortality begins to increase seem to be higher in the warmest cities (Tel-Aviv, Valencia and Tunis) and the steepest right-hand curves were observed in Athens, Barcelona, Bari, Lisbon and Rome.

**Figure 2:**
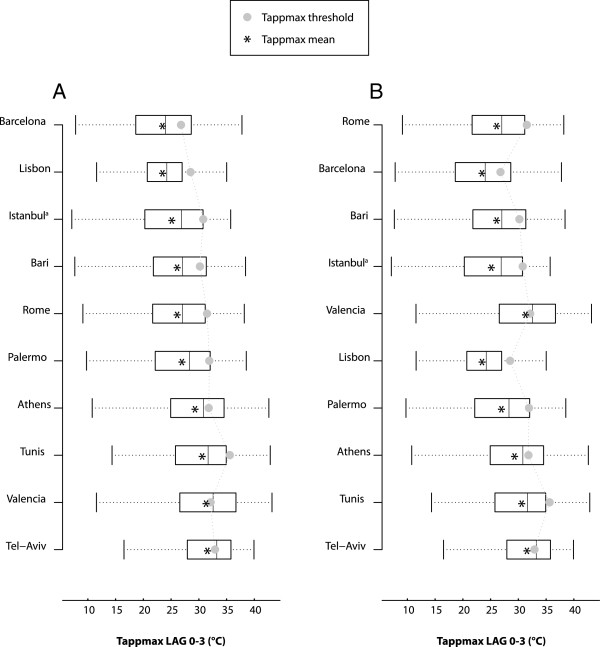
**Summer Tappmax (°C) distributions and threshold values by mean Tappmax (A) and latitude (B), **^**a **^**Mean apparent temperature.**

Figure 2 shows the summer Tappmax distributions and thresholds values in the study cities ordered by mean of summer Tappmax and by latitude. Threshold rises as the mean of summer Tappmax increases (part A), with higher values in the Eastern-Southern Mediterranean cities (Tunis and Tel-Aviv). In Valencia and Tel-Aviv the threshold value is very close to the mean summer Tappmax value. For a group of cities located closer to the equator (from Lisbon to Tel-Aviv), figure (part B) shows an increase in threshold values as latitude decreases, while for the other cities the trend is less clear.

Table 2 shows the effect of Tappmax above threshold on daily mortality in different age groups (all ages, 0–14, 15–64, 65–74 and 75+).

**Table 2:** Percentage change in all causes mortality for 1°C increase above Tappmax threshold by age group

**City**^**a**^	**all ages**		**0-14 age group**		**15-64 age group**		**65-74 age group**		**75+ age group**	
	**% change**	**95% CI**	**% change**	**95% CI**	**% change**	**95% CI**	**% change**	**95% CI**	**% change**	**95% CI**
Rome	6.4	5.8 - 7.0	-0.1	-5.9 - 6.0	2.9	1.7 - 4.2	5.0	3.9 - 6.1	7.9	7.2 - 8.6
Barcelona	3.2	2.7 - 3.7	-4.2	-12.1 - 4.4	1.2	0.1 - 2.3	2.1	1.0 - 3.1	4.0	3.4 - 4.6
Bari^b^	5.2	3.6 - 6.8	—	— —	8.9	5.1 - 12.8	5.2	1.8 - 8.7	4.2	2.2 - 6.3
Istanbul^c^	2.4	0.6 - 4.1	—	— —	5.6	2.5 - 8.8	5.5	2.6 - 8.5	—	— —
Valencia	1.4	0.7 - 2.1	1.0	-7.2 - 9.9	0.5	-1.1 - 2.1	0.0	-1.5 - 1.5	2.1	1.1 - 3.0
Lisbon	8.8	7.5 - 10.2	6.2	-8.3 - 23.1	3.3	0.5 - 6.1	6.3	3.7 - 9.1	11.6	9.9 - 13.4
Palermo^b^	3.2	0.7 - 5.7	—	— —	-1.2	-7.2 - 5.3	6.5	1.5 - 11.7	3.3	0.2 - 6.5
Athens	3.5	3.0 - 4.0	-2.5	-7.9 - 3.2	1.3	0.2 - 2.5	3.0	2.0 - 3.9	4.3	3.7 - 5.0
Tunis	4.3	2.7 - 5.9	7.6	3.2 - 12.2	2.3	-0.4 - 5.2	5.4	2.2 - 8.7	4.6	1.8 - 7.5
Tel-Aviv	2.0	0.9 - 3.2	5.3	-3.2 - 14.5	4.4	1.7 - 7.1	2.2	-0.2 - 4.6	1.0	-0.3 - 2.3

^a^ Cities are ordered by latitude.

^b^ Convergence not achieved for 0–14 age group.

^c^ Data not available for 0–14 and 75+ age group.

Considering all ages, the highest increase in daily mortality was observed in Lisbon (8.8%, 95%CI 7.5-10.2) and in the Italian cities of Rome (6.4%, 95%CI 5.8-7.0) and Bari (5.2%, 95%CI 3.6-6.8), whereas the lowest effect was estimated in Valencia (1.4%, 95%CI 0.7-2.1) and Tel-Aviv (2.0%, 95%CI 0.9-3.2). Considering the different age groups, the effect was increasing with age in Rome, Barcelona, Lisbon, Athens, Valencia and Palermo, but in the latter two cities the effect reached the statistical significance only in the oldest age groups (75+ in Valencia and 65–74 and 75+ in Palermo). In Tunis, Bari and Tel-Aviv the effect was greater in the population younger than 65 years; in particular, in Tunis within the 0–14 age group, while in Bari and Tel-Aviv in the 15–64 age group. In Istanbul the effect was similar in 15–64 and 65–74 age groups being almost twice compared with all ages population.

Percent of deaths attributable to Tappmax exposure above threshold varied by cities and by age groups as found for estimates of percent increases (Additional file 1: Table S4). The greatest proportion of deaths attributable to temperature was observed in Lisbon in the 75+ age group (10.4%), in Bari in the 15–64 age group (8.2%), in Rome in the 75+ age group (7.3%) and Tunis in the 0–14 age group (7.1%).

In Table 3 were reported only the city characteristics identified as effect modifiers in the meta-regression analysis, explaining more than 10% of between-city variance and with p-value<0.20.

**Table 3:** **Effect**^**a **^**modification by city characteristics evaluated at the 25th and 75th percentile of the effect modifier distribution**

	**25th percentile**			**75th percentile**			**Statistical significance**	
**Effect modifier**^**b**^	**value**	**% change**	**90% CI**	**value**	**% change**	**90% CI**	**p-value**	**Between-city variance explained (%)**
Longitude (degrees minutes seconds)	E 2°9'32''	4.9	3.1 - 6.7	E 23°39'10''	3.2	1.5 - 4.9	0.180	10.4
Minimum Tappmax (°C)	19.1	4.8	3.2 - 6.4	22.8	3.2	1.6 - 4.9	0.157	20.3
Maximum Tappmax (°C)	40.5	5.0	3.6 - 6.3	46.8	2.9	1.6 - 4.3	0.034	45.7
Mean Tappmax (°C)	29.8	4.8	3.5 - 6.2	34.5	2.6	1.0 - 4.3	0.047	36.0
Standard deviation of Tappmax (°C)	6.5	3.4	2.0 - 4.8	6.8	3.3	1.9 - 4.8	0.095	23.6
Mean of mean temperature (°C)	22.8	4.9	3.1 - 6.7	25.3	3.8	2.4 - 5.1	0.185	13.3
Standard deviation of relative humidity (%)	7.6	2.6	0.9 - 4.3	10.8	4.9	3.5 - 6.3	0.053	32.7
Health expenditure (% of GDP)	7.2	3.7	2.3 - 5.1	8.2	4.6	3.1 - 6.1	0.180	13.0
Unemployment (% of total labor force)	9.3	4.2	2.9 - 5.5	10.5	3.7	2.3 - 5.1	0.150	17.1

^a^ Effect estimates expressed as percentage change in all causes mortality for 1°C increase above Tappmax threshold.

^b^ Climate variables are calculated over the summer season (April-September).

Most of the effect modifiers identified were climatic variables while among the socio-demographic characteristics considered only health expenditure and unemployment resulted statistically significant. The effect modifier that explained the greatest proportion of between-city variance (45.7%) was the maximum Tappmax, while the other variables explained from 36.0% (mean Tappmax) to 10.4% (longitude).

The comparison of the predicted percent changes in mortality evaluated at 25^th^ and 75^th^ percentile shows how each city characteristic modifies the effect of Tappmax on mortality. Considering temperature variables (Tappmax, mean temperature), the predicted effect is greater among the cities with a milder climate. For example, in a city with a mean Tappmax of 29.8°C (25th percentile) mortality increased +4.8% while in a city with a mean Tappmax of 34.5°C (75th percentile) the increase was +2.6%. The predicted effect was slightly lower in cities with a greater standard deviation of Tappmax. For standard deviation of relative humidity we observed the opposite, with larger effects predicted in cities with greater variability in humidity values. The predicted effect was greater in cities with lower unemployment, higher health expenditure, and those further at West.

### Results from sensitivity analysis

City-specific results were robust to different modeling choices regarding the correlation structure, confounders, seasonal and long time trend. Also the thresholds estimation was robust to different starting points. Meta-regression analysis rerun with extreme threshold identified the same effect modifiers (results available on request).

## Discussion

The present study is the first to investigate the health impact of summer temperatures around the Mediterranean basin, including cities from both Europe for which some evidence is available [3-5], as well as Eastern-Southern Mediterranean cities. Cities cover a total population of 20 million people and a wide range of local climate conditions and socio-demographic characteristics. In our study, the effect of summer temperatures on all ages population was lower in the warmest Eastern-Southern cities than in the milder European cities. Previous studies found the opposite with the greatest effect among the warmest cities [3,5]. However, while in the European cities (except Bari) the greatest effect is recorded in the elderly, in the Eastern-Southern cities the greatest effect is among the youngest age groups (0–14 and 15–64 years).

A possible explanation of the different results by age group in our study could be searched in the different age structure between European and Eastern-Southern Mediterranean cities. In fact, the latter cities are characterized by a proportion of elderly below the European cities and this might explain the lower effect observed in this age group. On the contrary, the strong effect in young population underlines the higher vulnerability of this subgroup that represents a meaningful portion of population in these cities, almost double compared to European ones.

The higher vulnerability of older individuals during extreme summer temperatures is well known and can be attributable to the reduced thermoregulatory responses in these subjects and, in some cases, to the presence of chronic diseases, limited mobility and not being self-sufficient [28]. Considering youngest age groups previous studies conducted in developing countries have already observed a greater risk in the 0–14 years old group [29-31]. Physiological studies have shown that children may be more vulnerable, especially newborns, since they have an inefficient thermoregulatory response and are not able to care for themselves in particular concerning fluid intake or actions to reduce their exposure to heat [32,33]. In addition, in some Eastern-Southern Mediterranean countries the effect of summer temperature on young populations is expected to be magnified due to the higher occurrence of infectious diseases associated to the warmer climate and socioeconomic inequalities in these areas [34] that hamper capacity to adapt to extreme climate conditions [6].

In the adult population, the effect of summer temperature is relatively uncommon, unless in developed countries. Therefore results in Bari and Tel-Aviv regarding the effect among the 15–64 year olds are difficult to interpret, although there is some evidence that in this age group the presence of specific chronic diseases or specific occupational risks may increase vulnerability to heat [35]. However, this cannot be directly deduced from our analysis.

Our results suggest that in the warmest cities local populations may have developed a certain degree of acclimatization to summer temperatures. In fact, the high threshold values recorded in Tunis, Tel-Aviv and Valencia underline that in these cities only very high temperatures may still have an effect on mortality. On the other hand, it should be noted that in Tel-Aviv and Valencia where the threshold is very close to the mean Tappmax value, also less extreme temperatures contribute to the estimated effects that actually are the lowest compared with the other cities. According to previous studies, threshold values are influenced by local climatic conditions as well by latitude and local resources in place to prevent health effects related to temperature extremes [5,12]. In this study, the most important factor explaining differences in threshold value among cities was represented by local climate conditions, while we observe a weaker association with latitude in contrasts with previous studies [5,12] . This different result has no easy interpretation but it is worth considering that the study cities are located in a narrow range of latitude points and the limited variability may hide the association.

Local climatic conditions were also the most important explanatory factor for the large heterogeneity in the effect of Tappmax between Eastern-Southern and European Mediterranean cities. In particular, we observed higher effects among cities with milder temperature (Tappmax and mean temperature) and lower variability in Tappmax. Due to physiological acclimatization, in these populations the milder or less variable climate determines a limited tolerability to extreme temperatures outside the normal range. Multicenter studies from the US found similar results, with greater summer effects in populations with milder temperatures [9,10,12,36].

In the present study none of the socio-demographic and economic indicators was found to be a significant effect modifier of Tappmax-mortality relationship, except health expenditure and unemployment rate. A greater effect was observed in cities with a higher health expenditure that are those of more affluent countries. Similar findings were shown in US cities [10], while a study in Taiwan found the opposite [37]. In general the health expenditure is higher in developed countries where most of health costs derive from the higher proportion of elderly in the population, therefore the effect observed could be an artifact due to the higher prevalence of elderly population in European cities. A similar pattern of effect modification was found also for unemployment rate, with highest Tappmax effects in cities with lower value of this indicator; this result is difficult to interpret but is coherent with a previous US study [10]. Other socioeconomic and cultural characteristics, such as race, educational level, deprivation and religious group, can provide a more extensive explanation of the differences in the effect of Tappmax between European and non-European cities of the Mediterranean basin; however, no information on these factors was retrieved in the present study. On the other hand, in contrast with other studies [5,9,10], demographic characteristics as age structure and population density did not explain the between-city heterogeneity.

In the present study we estimated the effect of Tappmax using a V-shaped model with thresholds estimated using likelihood methods [24]. In other studies different approaches were used to estimate the temperature impact on mortality regarding the modeling of the exposure-response curve, lag structure and temperature metric [10]. Different approaches provide different results and hamper between-studies comparability. Actually, no gold standard modeling approach exists but it should be driven by the type of effect to be estimated. In our case, we wanted to produce city-specific estimates of the overall effect of summer temperature instead of estimating the effect of extreme temperatures; and in fact, the V-shaped model allows all days to contribute to the estimation of the temperature-mortality effects [20]. The robustness of our results regarding the overall effect of summer temperature was evaluated throughout a sensitivity analysis considering the effect in the range of extreme temperatures (from the 95th percentile) and the same pattern of mortality predictors among city-specific characteristics was identified, therefore excluding that effect estimates could have been biased due to the different choice of temperature threshold values.

More complex nonlinear functions have been used in alternative to the linear threshold model but it has been suggested that they are more suitable when comparing a large range of climates [10]; this is not the case of the present study where only two major climate types can be identified: European cities and Istanbul characterized by typical Mediterranean climates; while Tunis and Tel-Aviv characterized by a hot desert climate.

In the present study we estimated the effects of summer temperature by using maximum apparent temperature as temperature metric. This indicator was validated in previous European multicentre studies as being able to capture both temperature and humidity effects in a single parameter [3,4]. Other studies have adopted different temperature indicators (minimum, maximum, mean temperature, and other composite indexes such as humidex), but recent assessments found that no one temperature metric was superior to the others as predictor of mortality [38,39].

As already demonstrated by previous studies [3,4], a lag structure up to 3 days is able to capture the short-term effects of summer temperature on mortality suggesting a rapid physical response. In the European cities evidence of harvesting effect was found (results available on request), thus the actual effect of Tappmax calculated at lag 0–3 is probably overestimated because the amount of mortality displacement was not taken into account. Conversely, no harvesting effect was observed in the Eastern-Southern Mediterranean cities where the effect of summer temperature is therefore associated with a substantial loss in terms of years of life.

In the meta-regression analysis, only few characteristics were found to be significant effect modifiers of the summer temperature effect and this could be attributable to the fact that the available indicators were referred to the national level, thus not appropriately reflecting city-level characteristics. Moreover, the limited number of study cities may have reduced the statistical power and therefore limited the ability to identify the effect modifiers as discussed also in another multicentre study [13]. Although we included only three cities from the East and South of the Mediterranean (Tunis, Istanbul and Tel-Aviv) and time series for Istanbul and Tel-Aviv were dated, we argue that results from these cities could be used as comparison in future studies conducted also in cities with similar climatic, demographic and socioeconomic conditions. However, future research should be extended to include more cities from these areas, with particular focus on city-level socioeconomic characteristics that are important determinants of the adaptive capacity of local populations.

## Conclusions

The present study has provided for the first time evidence of the effect of summer temperature for the Mediterranean region including cities from North-Africa and the Middle East never studied before. Results indicate that this area urges more research and public health attention because of the high vulnerability of the youngest populations and the low level of economic resources that limit population capacity to adapt to extreme climate.

At the same time, it is worth noting that this combined effect of climatic and socio-demographic determinants, will probably further enlarge the fraction of vulnerable population in the Eastern-Southern Mediterranean area, since large political and economic changes are already occurring [6], as well as summer weather conditions are expected to get worse due to climate change [2].

For the same reasons, future studies should mainly focus on the south of the Mediterranean including a larger number of cities, to identify the major determinants of susceptibility to summer temperature at population level, as well also to monitor them over time.

## Abbreviations

EU: European Union; CIRCE: Climate change and impact research: the Mediterranean environment; US: United States; GDP: Gross domestic product; ICD-9: International classification of diseases, 9th revision; Tappmax: Maximum apparent temperature (°C); Temp: Air temperature (°C); Dew: Dew point temperature (°C); WHO: World Health Organization; OECD: Organisation for economic co-operation and development; GEEs: Generalized estimating equations; GAMs: Generalized additive models; QIC: Quasi-likelihood under the independence model criterion; NO2: Nitrogen dioxide; PM10: Particulate matter with an aerodynamic diameter of 10 μm or less.

## Competing interests

The authors declare that they have no competing interests.

## Authors’ contributions

ML designed the study and performed the statistical analysis and contributed to the draft of the manuscript. DD conceived, coordinated and designed the study, and drafted the manuscript. MDS helped draft the manuscript. AA contributed to the statistical analysis and provided the data for Athens. BM is an expert in public health from the WHO, the coordinator of Research Line 9 - “Human Health” of the CIRCE project with ABS. KK provided expert advise on the study design and was leader of WP9.2. FKdD contributed to the meteorological database, discussion of results and draft of the manuscript. XB provided the data for Barcelona. ABS provided the data for Tunis and was the coordinator of Research Line 9 - with BM. EC provided the data for Lisbon. ZD provided the data for Istanbul. CI provided the data for Valencia. CP provided the data for Tel-Aviv. TW collaborated in coordinating the Research Line 9 of the CIRCE project. PM conceived and supervised the study and helped to draft the manuscript. All authors read and approved the final manuscript.

## Supplementary Material

Additional file 1: Table S1 City-level population data sources. **Table S2:** Country-level demographic, socioeconomic and health care indicators data sources. **Table S3:** Study period, descriptive statistics of meteorological variables and Tappmax thresholds. **Table S4:** Percent of deaths attributable to temperatures above Tappmax city-threshold, by age group. Click here for file
