# Supplementary material for: A time series study on the effects of heat on mortality and evaluation of heterogeneity into European and Eastern-Southern Mediterranean cities: results of EU CIRCE project
Source: Environ Health. 2013 Jul 3;12:55. doi: 10.1186/1476-069X-12-55 (PMC3716565; doi:10.1186/1476-069X-12-55)
Supplement: Additional file 1: Table S1 — City-level population data sources. Table S2: Country-level demographic, socioeconomic and health care indicators data sources. Table S3: Study period, descriptive statistics of meteorological variables and Tappmax thresholds. Table S4: Percent of deaths attributable to temperatures above Tappmax city-threshold, by age group. [file 1476-069X-12-55-S1.doc]

Table S1. City-level population data sources.

| City | Year | Source | URL |
| --- | --- | --- | --- |
| Rome | 2001 | National Institute of Statistics, Italy | http://www.urbanaudit.org |
| Barcelona | 2001 | Instituto Nacional de Estadística, Madrid | http://www.urbanaudit.org |
| Bari | 2001 | National Institute of Statistics, Italy | http://www.urbanaudit.org |
| Istanbul | 2001 | State Institute of Statistics, Republic of Turkey | http://www.urbanaudit.org |
| Valencia | 2001 | Instituto Nacional de Estadística, Madrid | http://www.ine.es/ |
| Lisbon | 2001 | Instituto Nacional de Estatística Portugal | http://www.urbanaudit.org |
| Palermo | 2001 | National Institute of Statistics, Italy | http://www.urbanaudit.org |
| Athens | 2001 | National Statistical Service of Greece | http://www.urbanaudit.org |
| Tunis | 2004 | Institut National de la Statistique Tunisie | www.ins.nat.tn/indexen.php |
| Tel-Aviv | 1995 | Central Bureau of Statistics | http://www1.cbs.gov.il/census/census/pnimi_page_e.html?id_topic=3 |

Table S2. Country-level demographic, socioeconomic and health care indicators data sources.

|  | Infant mortality rates (per 1 000 live births) | |  | Life expectancy at birth (years) | | |  | Hospital beds Density x 1000 | |  | GDP per capita (US $) | |  | Health expenditure (% of GDP) | |  | Unemployment total (% of total labor force) | |
| --- | --- | --- | --- | --- | --- | --- | --- | --- | --- | --- | --- | --- | --- | --- | --- | --- | --- | --- |
| Country | Year | Source |  | | Year | Source |  | Year | Source |  | Year | Source |  | Year | Source |  | Year | Source |
| Italy | 2001 | OECD^a^ |  | | 2001 | OECD^a^ |  | 2001 | OECD^a^ |  | 2001 | World Bank^c^ |  | 2001 | World Bank^c^ |  | 2001 | World Bank^c^ |
| Spain | 2001 | OECD^a^ |  | | 2001 | OECD^a^ |  | 2001 | OECD^a^ |  | 2001 | World Bank^c^ |  | 2001 | World Bank^c^ |  | 2001 | World Bank^c^ |
| Turkey | 2001 | OECD^a^ |  | | 2001 | OECD^a^ |  | 2001 | OECD^a^ |  | 2001 | World Bank^c^ |  | 2001 | World Bank^c^ |  | 2001 | World Bank^c^ |
| Portugal | 2001 | OECD^a^ |  | | 2001 | OECD^a^ |  | 2001 | OECD^a^ |  | 2001 | World Bank^c^ |  | 2001 | World Bank^c^ |  | 2001 | World Bank^c^ |
| Greece | 2001 | OECD^a^ |  | | 2001 | OECD^a^ |  | 2001 | OECD^a^ |  | 2001 | World Bank^c^ |  | 2001 | World Bank^c^ |  | 2001 | World Bank^c^ |
| Tunisia | 2004 | WHO^b^ |  | | 2001 | World Bank^c^ |  | 2002 | WHO^d^ |  | 2001 | World Bank^c^ |  | 2001 | World Bank^c^ |  | 2001 | World Bank^c^ |
| Israel | 2001 | OECD^a^ |  | | 2002 | OECD^a^ |  | 2002 | OECD^a^ |  | 2001 | World Bank^c^ |  | 2001 | World Bank^c^ |  | 2001 | World Bank^c^ |

^a^ OECD, OECD Statistics extracts (database) [16]

^b^ WHO, World Health Statistics 2006 [19]

^c^ The World Bank, World Development Indicators (database) [15]

^d^ WHO, World Health Statistics 2005 [17]

Table S3. Study period, descriptive statistics of meteorological variables and Tappmax thresholds

| **City**^a^ | **Study period** | **Tappmax (°C)** | |  | **Mean Temperature (°C)** | |  | **Minimum Temperature (°C)** | |  | **Maximum Temperature (°C)** | |  | **Relative Humidity (%)** | |  | **Tappmax threshold (°C)**^b^ | **95%CI** | | |
| --- | --- | --- | --- | --- | --- | --- | --- | --- | --- | --- | --- | --- | --- | --- | --- | --- | --- | --- | --- | --- |
|  |  | **Mean** | **SD** |  | **Mean** | **SD** |  | **Mean** | **SD** |  | **Mean** | **SD** |  | **Mean** | **SD** |  |  |  |  |  |
| **Rome** | 1992-2006 | 26.2 | 6.5 |  | 20.6 | 4.7 |  | 16.0 | 4.5 |  | 25.4 | 5.4 |  | 50.2 | 10.1 |  | 31.4 | 31.1 | - | 31.7 |
| **Barcelona** | 1991-2004 | 23.5 | 6.8 |  | 19.5 | 4.8 |  | 16.9 | 4.7 |  | 22.8 | 5.3 |  | 68.8 | 12.2 |  | 26.7 | 26.2 | - | 27.3 |
| **Bari** | 1996-2004 | 26.1 | 6.8 |  | 21.0 | 4.8 |  | 16.7 | 4.7 |  | 24.9 | 5.2 |  | 68.6 | 10.8 |  | 30.1 | 29.0 | - | 31.2 |
| **Istanbul** | 1992-1995 | 25.3^c^ | 7.0^c^ |  | 19.6 | 4.9 |  | - | - |  | -- | -- |  | 75.2 | 8.3 |  | 30.7 | 29.8 | - | 31.6 |
| **Valencia** | 1994-2003 | 31.4 | 6.9 |  | 22.4 | 4.2 |  | 19.0 | 4.3 |  | 26.3 | 4.5 |  | 62.5 | 12.4 |  | 32.0 | 29.3 | - | 34.7 |
| **Lisbon** | 2000-2004 | 23.5 | 5.1 |  | 20.0 | 3.9 |  | 16.5 | 3..3 |  | 23.8 | 4..6 |  | 63.9 | 13.3 |  | 28.4 | 28.0 | - | 28.8 |
| **Palermo** | 2001-2005 | 27.0 | 6.5 |  | 19.7 | 4.0 |  | 20.9 | 4.7 |  | 25.9 | 5.0 |  | 51.2 | 9.7 |  | 31.8 | 30.7 | - | 33.0 |
| **Athens** | 1997-2004 | 28.6 | 6.8 |  | 23.9 | 5.2 |  | 20 | 4.9 |  | 28.4 | 5.7 |  | 55.5 | 11.9 |  | 31.7 | 31.2 | - | 32.2 |
| **Tunis** | 2005-2007 | 30.7 | 6.5 |  | 22.5 | 5.0 |  | 20.9 | 4.9 |  | 29.9 | 5.5 |  | 63.0 | 11.4 |  | 35.5 | 33.7 | - | 37.3 |
| **Tel-Aviv** | 1991-1996 | 31.7 | 5.7 |  | 23.9 | 3.6 |  | 20.2 | 3.9 |  | 27.7 | 3.7 |  | 66.5 | 8.6 |  | 32.8 | 31.8 | - | 33.9 |

^a^ Cities are ordered by latitude

^b^ Tappmax (°C, lag 0-3) thresholds estimated by segmented regression [24]

^c^ Mean apparent temperature

Table S4. Percent of deaths attributable to temperatures above Tappmax city-threshold, by age group

| **City** ^a^ | **all ages** | | | |  | **0-14 age group** | | | |  | **15-64 age group** | | | |  | **65-74 age group** | | | |  | **75+ age group** | | | |
| --- | --- | --- | --- | --- | --- | --- | --- | --- | --- | --- | --- | --- | --- | --- | --- | --- | --- | --- | --- | --- | --- | --- | --- | --- |
|  | **% attributable deaths** | **95% CI** | | |  | **% attributable deaths** | **95% CI** | | |  | **% attributable deaths** | **95% CI** | | |  | **% attributable deaths** | **95% CI** | | |  | **% attributable deaths** | **95% CI** | | |
| Rome | 6.0 | 5.5 | - | 6.5 |  | -0.1 | -6.3 | - | 5.7 |  | 2.8 | 1.7 | - | 4.0 |  | 4.8 | 3.8 | - | 5.7 |  | 7.3 | 6.7 | - | 7.9 |
| Barcelona | 3.1 | 2.6 | - | 3.6 |  | -4.4 | -13.8 | - | 4.2 |  | 1.2 | 0.1 | - | 2.2 |  | 2.1 | 1.0 | - | 3.0 |  | 3.8 | 3.3 | - | 4.4 |
| Bari^b^ | 4.9 | 3.5 | - | 6.4 |  | --- | --- |  | --- |  | 8.2 | 4.9 | - | 11.3 |  | 4.9 | 1.8 | - | 8.0 |  | 4.0 | 2.2 | - | 5.9 |
| Istanbul^c^ | 2.3 | 0.6 | - | 3.9 |  | --- | --- |  | --- |  | 5.3 | 2.4 | - | 8.1 |  | 5.2 | 2.5 | - | 7.8 |  | --- | --- |  | --- |
| Valencia | 1.4 | 0.7 | - | 2.1 |  | 1.0 | -7.8 | - | 9.0 |  | 0.5 | -1.1 | - | 2.1 |  | 0.0 | -1.5 | - | 1.5 |  | 2.1 | 1.1 | - | 2.9 |
| Lisbon | 8.1 | 7.0 | - | 9.3 |  | 5.8 | -9.1 | - | 18.8 |  | 3.2 | 0.5 | - | 5.7 |  | 5.9 | 3.6 | - | 8.3 |  | 10.4 | 9.0 | - | 11.8 |
| Palermo^b^ | 3.1 | 0.7 | - | 5.4 |  | --- | --- |  | --- |  | -1.2 | -7.8 | - | 5.0 |  | 6.1 | 1.5 | - | 10.5 |  | 3.2 | 0.2 | - | 6.1 |
| Athens | 3.4 | 2.9 | - | 3.8 |  | -2.6 | -8.6 | - | 3.1 |  | 1.3 | 0.2 | - | 2.4 |  | 2.9 | 2.0 | - | 3.8 |  | 4.1 | 3.6 | - | 4.8 |
| Tunis | 4.1 | 2.6 | - | 5.6 |  | 7.1 | 3.1 | - | 10.9 |  | 2.2 | -0.4 | - | 4.9 |  | 5.1 | 2.2 | - | 8.0 |  | 4.4 | 1.8 | - | 7.0 |
| Tel-Aviv | 2.0 | 0.9 | - | 3.1 |  | 5.0 | -3.3 | - | 12.7 |  | 4.2 | 1.7 | - | 6.6 |  | 2.2 | -0.2 | - | 4.4 |  | 1.0 | -0.3 | - | 2.2 |

^a^ Cities are ordered by latitude

^b^ Convergence not achieved for 0-14 age group

^c^ Data not available for 0-14 and 75+ age group
